# Diverse proinflammatory response in pharyngeal epithelial cells upon interaction with *Neisseria meningitidis* carriage and invasive isolates

**DOI:** 10.1186/s12879-024-09186-3

**Published:** 2024-03-06

**Authors:** Alexander Persson, Therese Koivula, Susanne Jacobsson, Bianca Stenmark

**Affiliations:** 1 School of Medical Sciences, Faculty of Medicine and Health, Örebro University, Örebro, Sweden; 2 Inflammatory Response and Infection Susceptibility Centre (iRiSC), Faculty of Medicine and Health, Örebro University, Örebro, Sweden; 3 Department of Laboratory Medicine, Faculty of Medicine and Health, Örebro University, Örebro, Sweden

**Keywords:** IMD, FaDu, Cytokines, Chemokines, Cell death, Adhesion, Host pathogen interaction

## Abstract

**Background:**

Invasive meningococcal disease (IMD), including sepsis and meningitis, can develop when *Neisseria meningitidis* bacteria breach the barrier and gain access to the circulation. While IMD is a rare outcome of bacterial exposure, colonization of the oropharynx is present in approximately 10% of the human population. This asymptomatic carriage can be long or short term, and it is unknown which determining factors regulate bacterial colonization. Despite descriptions of many bacterial virulence factors and recent advances in detailed genetic identification and characterization of bacteria, the factors mediating invasion and disease vs. asymptomatic carriage following bacterial colonization remain unknown. The pharyngeal epithelia play a role in the innate immune defense against pathogens, and the aim of this study was to investigate the proinflammatory response of pharyngeal epithelial cells following meningococcal exposure to describe the potential inflammatory mediation performed during the initial host‒pathogen interaction. Clinically relevant isolates of serogroups B, C, W and Y, derived from patients with meningococcal disease as well as asymptomatic carriers, were included in the study.

**Results:**

The most potent cellular response with proinflammatory secretion of TNF, IL-6, CXCL8, CCL2, IL-1β and IL-18 was found in response to invasive serogroup B isolates. This potent response pattern was also mirrored by increased bacterial adhesion to cells as well as induced cell death. It was, however, only with serogroup B isolates where the most potent cellular response was toward the IMD isolates. In contrast, the most potent cellular response using serogroup Y isolates was directed toward the carriage isolates rather than the IMD isolates. In addition, by comparing isolates from outbreaks in Sweden (epidemiologically linked and highly genetically similar), we found the most potent proinflammatory response in cells exposed to carriage isolates rather than the IMD isolates.

**Conclusion:**

Although certain expected correlations between host‒pathogen interactions and cellular proinflammatory responses were found using IMD serogroup B isolates, our data indicate that carriage isolates invoke stronger proinflammatory activation of the epithelial lining than IMD isolates.

**Supplementary Information:**

The online version contains supplementary material available at 10.1186/s12879-024-09186-3.

## Background

*Neisseria meningitidis* (the meningococcus) can cause invasive meningococcal disease (IMD) with high morbidity and mortality through epidemic or sporadic meningitis and/or septicaemia [1]. IMD results from a multifactorial process involving bacterial virulence factors, environmental factors and host susceptibility factors, bacterial exposure, colonization, invasion and survival [2, 3]. *N. meningitidis* is an obligate human pathogen with the oropharynx as the only niche, and an estimated 10% of the general healthy population are asymptomatic carriers, although carrier rates ranging from 1 to 40% have been reported [4–10]. Asymptomatic carriage has been shown to extend as long as 12 months, but since no data are available for a longer time, it is unclear how protracted meningococcal carriage can be [6]. No clear correlation has further been shown between individual carriage and IMD, indicating that carriage may not be the main cause of progression into invasive disease. Rather, disease cases most likely occur upon new contraction of the pathogen. Despite the prevalence of meningococcal carriage in populations worldwide and recent advances in molecular microbiology, it has been difficult to pinpoint the reason for a certain meningococcal strain to progress to invasive disease or merely lead to asymptomatic colonization. Host‒pathogen interactions at the site of colonization are likely to regulate carriage, restrict bacterial growth and restrain bacterial invasion.

The airway mucus barrier restricts meningococcal colonization, and the epithelial cell lining in the pharyngeal mucosa has the capacity to actively regulate the microenvironment upon bacterial sensing [11]. It is well accepted that epithelial cells constitute more than a mechanical barrier between the external environment and the underlying mesenchyme. These cells have the capacity to recognize and respond to microbes through mechanisms shared with cells of the innate immune system, including pattern recognition receptors (PRRs) and intracellular nucleotide-binding domain leucine-rich repeat containing receptors (NLRs) [12, 13]. Upon recognition of noxious stimuli such as bacteria, epithelial cells effectively mediate inflammatory signaling to maintain homeostasis and facilitate immune responses. Epithelial cells respond to meningococci with a barrage of inflammatory modulators, including both direct bactericidal peptides such as LL-37 and the production and release of cytokines and chemokines involved in the recruitment and activation of immune cells [14–18]. In addition to canonical production and release of cytokines, IL-1β and IL-18 of the IL-1 family require processing by a serine protease to become biologically active [19, 20]. This is mediated by NLRs such as NLRP3, which initiate the composition of cytoplasmic multiprotein complexes called inflammasomes. LOS is a meningococcal virulence factor, and we have previously shown that human innate immune cells respond to meningococcal LOS sensing with potent activation of the NLRP3 inflammasome [21, 22]. Whether this cellular activation is host- or bacteria-beneficial is yet to be discerned since this proinflammatory system, in addition to mediating a strong anti-pathogenic inflammatory environment with immune cell activation, is also capable of destroying cells in lytic cell death, which may facilitate invasion. In addition to the induced production and release of cytokines, the IL-1 family member IL-33 is constitutively produced by epithelia in structural and lining barriers [23]. IL-33 is released upon tissue damage, e.g., during bacterial infection, suggesting a role as an alarmin capable of alerting resident immune cells to mount an immunocompetent response [23].

Meningococci are classified into twelve capsular groups depending on the polysaccharide composition of the capsule, of which serogroups A, B, C, W, Y and X are the most common in clinical circumstances [24]. Furthermore, molecular typing using multilocus sequence typing (MLST) classifies meningococcus into different sequence types (STs) and clonal complexes (cc) based on polymorphisms in seven housekeeping genes [25]. Carriage rates are lower during nonendemic periods compared to high endemic periods, and, historically, serogroup B has been the dominant disease-causing serogroup [5, 26, 27]. Monitoring the dynamics of meningococcal disease in Sweden, an increase in invasive serogroup Y in 2006–2019 (Eriksson et al. unpublished) was followed by a high prevalence of Y among healthy carriers in the population in 2018–2019 [6]. In contrast, the increase in invasive W cases in 2015–2020 (Erikson et al. unpublished) was not followed by any increase in healthy carriage [6]. These observations clearly point out the dynamic nature of meningococcal exposure and how it may cause invasive disease or asymptomatic colonization. Whether the outcome is directed by bacterial virulence factors or the host response regulating bacterial colonization is still unknown.

The aim of the present study was to investigate the inflammatory response in human pharyngeal epithelial cells that may influence asymptomatic carriage when exposed to clinically relevant meningococcal isolates of both IMD and asymptomatic carriage.

## Results

### Difference in inflammatory response between serogroups

**Fig. 1:**
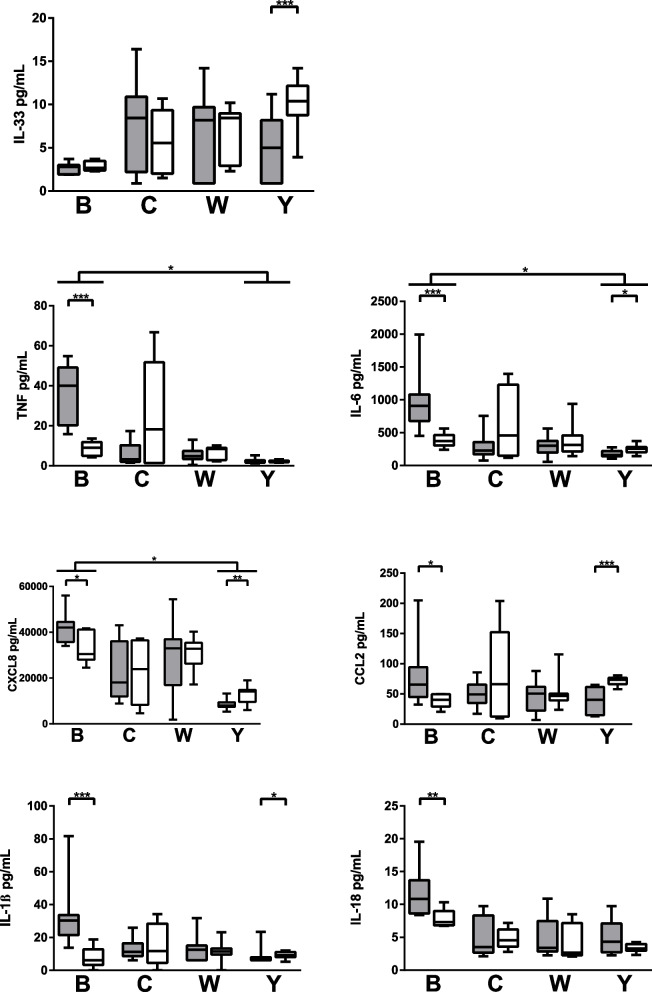
Cytokine and chemokine levels from epithelial cells exposed to *N. meningitidis* of invasive (gray) and carriage (white) isolates of serogroups B (invasive *n* = 2, carriage *n* = 1), C (invasive *n* = 3, carriage *n* = 2), W (invasive *n* = 7, carriage *n* = 3) and Y (invasive *n* = 2, carriage *n* = 2). Box plots represent the median, 1st and 3rd quartiles and min/max from 6 experiments for each isolate. Differences between serogroups were calculated using one-way ANOVA using Kruskal‒Wallis testing and Dunn’s multiple comparison corrections, and differences between invasive and carriage isolates within each serogroup were calculated using a Mann‒Whitney test

Of the 13 cytokines and chemokines in the LEGENDplex panel, 7 analytes (CCL2, CXCL8, TNF, IL-6, IL-1β, IL-18 and IL-33) were quantifiable in our model of exposed FaDu cells. The cellular proinflammatory response with release of TNF (median ± IQR: 28 ± 36 vs 3 ± 2), IL-6 (743 ± 764 vs 179 ± 71), CXCL8 (36958 ± 13532 vs 9945 ± 5942) was more potent towards serogroup B compared to serogroup Y (Fig. 1). Furthermore, cells exposed to invasive isolates of serogroup B responded with an increased inflammatory response of TNF (40 ± 29 vs 9 ± 7), IL-6 (908 ± 405 vs 372 ± 158), CXCL8 (42113 ± 8818 vs 30480 ± 13152), CCL2 (66 ± 49 vs 40 ± 20), IL-1β (30 ± 13 vs 6 ± 10) and IL-18 (11 ± 5 vs.7 ± 2) compared to carriage isolates. In contrast, within serogroup Y, the more potent inflammatory response was generated by cells exposed to the carriage isolates for IL-33 (10 ± 3 vs. 5 ± 7), IL-6 (256 ± 77 vs. 159 ± 80), CXCL8 (14145 ± 5309), CCL2 (73 ± 11 vs. 40 ± 46) and IL-1β (9 ± 3 vs. 7 ± 1), although quantities are low as serogroup Y isolates generally generated a weak proinflammatory cell response throughout.

### Differences in the inflammatory response between invasive and carriage isolates

**Fig. 2:**
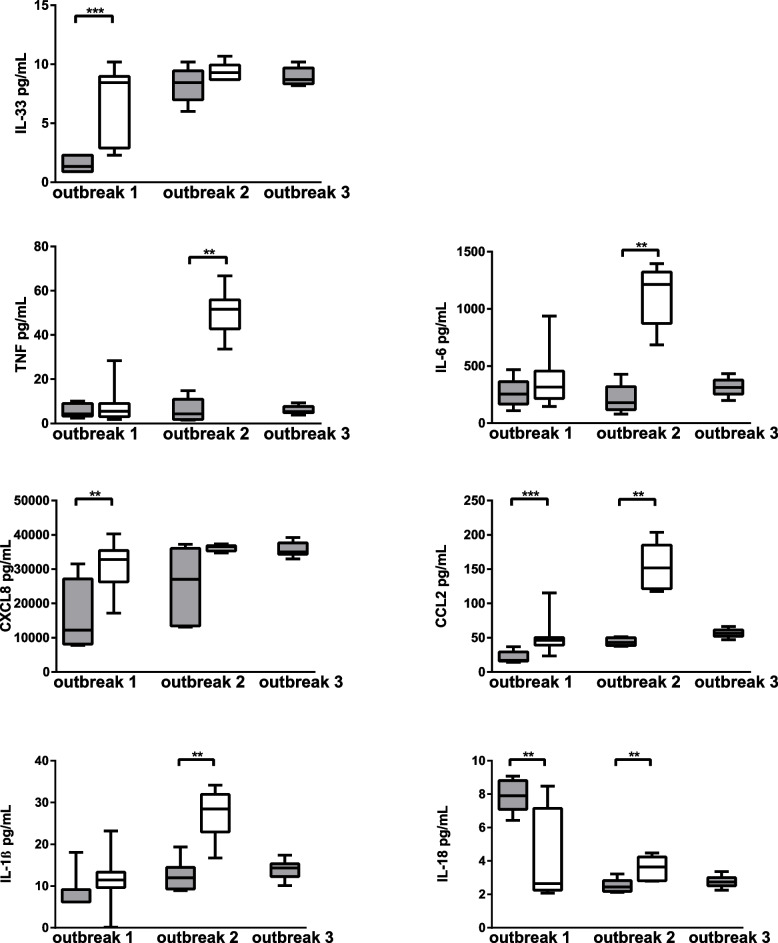
Cytokine and chemokine levels from epithelial cells exposed to *N. meningitidis* of IMD (gray) and carriage (white) isolates from outbreak 1 (invasive *n* = 1, carriage *n* = 3), outbreak 2 (invasive *n* = 1, carriage *n* = 1) and outbreak 3 (invasive *n* = 3). Box plots represent the median, 1st and 3rd quartiles and min/max from 6 experiments for each isolate. Differences between invasive and linked carriage isolates within each outbreak were calculated using a Mann‒Whitney test

To further investigate the host‒pathogen interaction with regards to cellular response, we compared carriage and IMD isolates within the same outbreak, i.e., isolates identified as highly similar using whole-genome sequencing (belonging to the same lineage, see Table 1 and Supplementary Figure 1) and isolated from individuals in close contact with the patients. These comparisons revealed that the epithelial cellular proinflammatory response with release of IL-33 (9 ± 7 vs. 2 ± 0), CXCL8 (32814 ± 9167 vs. 12245 ± 19014) and CCL2 (46 ± 11 vs. 17 ± 13) was more potent when exposed to the carriage isolates compared to the IMD counterpart from outbreak 1, serogroup W (Fig. 2). The linked isolates from outbreak 2 of serogroup C revealed similar results, where cells responded with TNF (52 ± 13 vs. 4 ± 9), IL-6 (1213 ± 450 vs. 179 ± 202), CCL2 (152 ± 63 vs. 43 ± 11), IL-1β (28 ± 9 vs. 12 ± 5) and IL-18 when exposed to the carriage isolate compared to the IMD-associated isolate. The cellular proinflammatory response within outbreak 3 was similar to all three invasive W isolates belonging to a Hajj lineage.

**Table 1:** Information regarding the isolates of *Neisseria meningitidis* used in the study

Study ID	Invasive vs. carriage	Capsular group	CC	ST	PorA VR	Lineage	Outcome	Outbreak	Ref
NM6	Invasive	B	cc32	32	P1.7,16		Fatal		[28]
NM18	Invasive	B	cc32	32	P1.7,16		Recovered		[28]
NM1	Invasive	C	cc11	11	P1.5,2		Fatal		[28]
NM23	Invasive	C	cc11	11	P1.5,2		Recovered		[28]
NM12	Invasive	C	cc32	32	P1.7,16–29		Fatal	Outbreak 2	[29]
NM21	Invasive	Y	cc23	23	P1.5–2,10–1	YI Subtype 1	Recovered		[30]
NM2	Invasive	Y	cc23	23	P1.5–2,10–1	YI Subtype 1	Recovered		[30]
NM11	Invasive	W	cc11	11	P1.5,2	Hajj	Recovered	Outbreak 3	[31]
NM14	Invasive	W	cc11	11	P1.5,2	Hajj	Fatal	Outbreak 3	[31]
NM17	Invasive	W	cc11	11	P1.5,2	Hajj	Recovered	Outbreak 3	[31]
NM19	Invasive	W	cc11	11	P1.5,2	Original UK	Fatal		[30]
NM24	Invasive	W	cc11	11	P1.5,2	UK 2013	Recovered		[30]
NM22	Invasive	W	cc11	11	P1.5,2	UK 2013	Recovered	Outbreak 1	[32]
NM7	Carriage	B	cc32	32	P1.7,16		NA		
NM8	Carriage	C	cc11	5116	P1.5,2		NA		
NM15	Carriage	C	cc32	32	P1.7,16–29		NA	Outbreak 2	[29]
NM3	Carriage	Y	cc23	23	P1.5–2,10–1	YI Subtype 1	NA		[33]
NM4	Carriage	Y	cc23	23	P1.5–2,10–1	YI Subtype 1	NA		[33]
NM9	Carriage	W	cc11	11	P1.5,2	UK 2013	NA	Outbreak 1	[32]
NM13	Carriage	W	cc11	11	P1.5,2	UK 2013	NA	Outbreak 1	[32]
NM16	Carriage	W	cc11	11	P1.5,2	UK 2013	NA	Outbreak 1	[32]
NM5	Ref. lab strain	W	cc11	11	P1.5,2	Hajj	NA		M7089B

*CC* Clonal complex, *ST* Sequence type, *VR* Variable region, *NA* Not available

### Association between adhesion and cell death

**Fig. 3:**
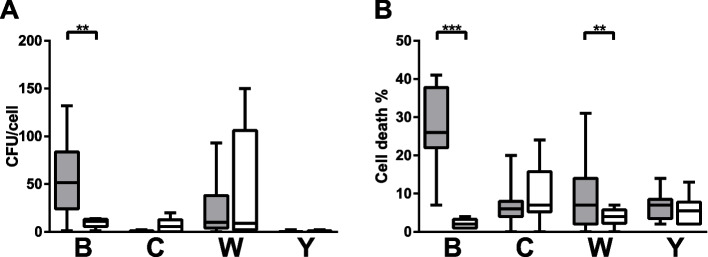
Adhesion (**A**) and cell death (**B**) in epithelial cells exposed to *N. meningitidis* of case (gray) and carriage (white) isolates of serogroups B (invasive *n* = 2, carriage *n* = 1), C (invasive *n* = 3, carriage *n* = 2), W (invasive *n* = 7, carriage *n* = 3) and Y (invasive *n* = 2, carriage *n* = 2). Box plots represent the median, 1st and 3rd quartiles and min/max from 5 experiments for each isolate. Differences between serogroups were calculated using one-way ANOVA using Kruskal‒Wallis testing and Dunn’s multiple comparison corrections, and differences between invasive and carriage isolates within each serogroup were calculated using a Mann‒Whitney test

Adhesion of bacteria to the epithelial lining is proposed as an important feature of colonization and potential invasion and immune evasion of meningococci. Many host‒pathogen interaction studies show that there is a strong correlation between the interaction and the cellular response in terms of proinflammatory activation. In our study, the IMD isolates of serogroup B presented increased adhesion and cell death compared to the carriage B isolate (52 ± 60 vs. 11 ± 7 for adhesion and 26 ± 4 vs. 2 ± 2 for cell death) (Fig. 3A and B). Cell death was also increased in cells exposed to the invasive W isolates compared to the carriage isolates (7 ± 12 vs. 4 ± 4) (Fig. 3B).

**Fig. 4:**
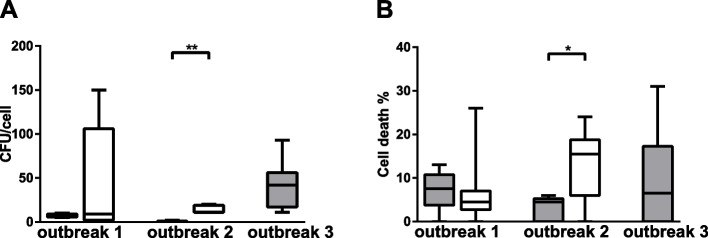
Adhesion (**A**) and cell death (**B**) in epithelial cells exposed to *N. meningitidis* of case (solid black) and carriage (open gray) isolates from outbreak 1 (invasive *n* = 1, carriage *n* = 3), outbreak 2 (invasive *n* = 1, carriage *n* = 1) and outbreak 3 (invasive *n* = 3). Box plots represent the median, 1st and 3rd quartiles and min/max from 5 experiments for each isolate. Differences between invasive and linked carriage isolates within each outbreak were calculated using a Mann‒Whitney test

In contrast, the carriage isolate from outbreak 2 resulted in more adhesion and cell death than the IMD isolate (11 ± 8 vs. 0 ± 0 for adhesion and 16 ± 13 vs. 5 ± 5 for cell death) in the outbreak (Fig. 4A and B). In order to study if the significant differences in adhesion between the invasive and linked carriage case in outbreak 2 could be explained by genetic differences, a genetic comparison of all defined loci in pubMLST (*n* = 3,136) was performed between the invasive and linked carriage isolates. The invasive isolate in Outbreak 2 lacked locus NEIS033, which encodes the type IV pilus associated protein and NEIS2099, which encodes a putative immune protein. NEIS2099 (alias NMB2106) is located in a possible operon coding for silent 3′ cassettes at the *maf-3* locus that has previously been reported as significantly downregulated on *Neisseria* attachment to the epithelial cells [34], suggesting these genes may play a role during bacterial adhesion.

Our data derived from the perspective of cellular responses allowed us to perform correlation analysis of our cellular readouts to the quantified adhesion of meningococci to the cells. Interestingly, as can be seen in Supplementary Figure 2 the overall correlation of adhesion with cell death was weak (*r* = 0.25), whereas the correlation between adhesion and the proinflammatory response mediators CXCL8, IL-6 and TNF was moderate (*r* = 0.79, 0.71 and 0.68, respectively).

## Discussion

Invasive disease is a rare outcome of encounters with meningococci and is proposed to occur shortly after acquisition of the bacterium by a susceptible host. The determining factors directing whether the host‒pathogen interaction will result in IMD, colonization and carriage or eradication of the bacteria are not well understood. The epithelial cell response to meningococci has been extensively investigated and provides insights into key mechanisms as well as the main bacterial traits involved. However, most studies have focused on the identification of bacterial traits involved in IMD, and most often few bacterial isolates are investigated without comparisons between different isolates. The colonization where the bacteria are allowed to remain but prevented from extensive expansion likely depends on a dynamic interplay between the colonizing bacteria and the host epithelial cells, and it is challenging to discern which mechanisms are initiated by the host and which are induced by the bacteria. In this study, we investigated the capacity of pharyngeal epithelial cells to mount a proinflammatory response following exposure to meningococcal isolates derived from clinical settings.

The soluble cytokines investigated here included the instructive cytokines IL-6 and TNF, which are often described in the context of meningococcal infections and exposures [35–37]. Our data indicate that whereas isolates from serogroups C, W and Y mediate a modest proinflammatory activation in the epithelial cells with regards to IL-6 and TNF, cells respond strongly to invasive B isolates where a marked interaction with cells and cell death was detected to a greater degree than the carriage isolate. In addition, the strongest cellular response with regards to IL-6 and TNF was found in cells exposed to the carriage isolate and not the IMD isolate. A similar cellular response was found with the inflammasome-associated cytokines IL-1β and IL-18 as well as the chemokine CCL2. Chemokines CCL2 and CXCL8 are central mediators of the recruitment of granulocytes and monocytes from circulation to the epithelial lining, where they participate in the first line of defense against pathogen invasion. Endothelial cells have been shown to release these chemokines upon recognition of one isolate of serogroup B meningococci [38]. Whereas no difference was found between all IMD and carriage W isolates used in this study with regard to the cell cytokine response, cells exposed to W isolates from outbreak 1 responded to a higher extent with chemokines CCL2 and CXCL8 as well as the alarmin IL-33 when exposed to the genetically similar carriage isolate compared to the IMD isolates. The cellular response to all three IMD isolates from outbreak 3 was similar. The conclusion from our comparisons of genetically similar (however few) isolates is quite surprising that the most potent proinflammatory cellular response in the epithelial cells is derived following exposure to the carriage isolates and not the IMD-associated isolates. We hypothesize that the carriage isolates may be kept in check due to the stronger proinflammatory response to their presence, whereas IMD isolates avoid inflammatory induction with less immune cell recruitment to the epithelial lining.

Although evidence regarding inflammasome involvement in *N. meningitidis* interaction with epithelial linings is largely lacking, *Neisseria* gonorrhoeae potently mediates lytic cell death in epithelial cells, a trait of inflammasome-mediated pyroptosis [39]. Host cell death plays an important role in the pathogenesis of several bacteria, and meningococci have been shown to both induce and prevent cell death [40–43]. To investigate the cell death aspect of meningococcal interaction, we studied cell viability following 24 h of host‒pathogen interaction. Similar to the strongest proinflammatory activation of cells as a response to the invasive B isolates, cell death was also induced to a larger extent by these isolates. In addition, cells exposed to the carriage C isolate from outbreak 2, which mediated the stronger proinflammatory response, showed cell death to a larger extent than the IMD isolate. The correlation between proinflammatory activity and cell death is not surprising but makes the distinction between the involved mechanisms challenging. In the current study, we cannot differentiate between the primary cellular response to meningococci and the secondary effects mediated by the cell through autocrine signaling, e.g., TNF is a known cell death inducer. It is logical to speculate that cell death is a trait beneficial for invasion through the epithelial lining, but our current data do not support this hypothesis. Since the invasive B isolates mediated stronger cell death, the opposite was found using our genetically similar outbreak isolates, and no clear conclusions can be drawn regarding the correlation between cell death and disease-causing capacity on the meningococcal isolates investigated. However, it is possible that a correlation cannot be made on a more general ‘invasive vs carriage’ level but only when comparing highly similar isolates eliciting different clinical outcomes.

Pathogen interaction with and adhesion to the host cell is often described as a main contributing factor to the inflammatory response by the cells. Several bacterial traits and molecular expressions facilitating adhesion to the host cells have been described [44–46]. Although many bacterial and host factors have been identified, several aspects of meningococcal pathogenesis remain unknown. No decisive expression pattern specifically associated with invasive isolates has been described, as several carriage isolates express adhesion molecules. Interestingly, despite the expression of the same surface molecules as disease-associated isolates, certain carriage isolates are less efficient in adherence and invasion for unknown reasons [47]. Out of all meningococcal isolates investigated in this study, 10 isolates were found to associate with cellular adhesion exceeding 10 CFU/cell following 6 h of incubation at an MOI of 10. Isolates belonging to serogroups B, C and W all entailed isolates that adhered to the epithelial cells in our model. Interestingly, despite the strong correlation in the societal presence of invasive and carriage serogroup Y in 2018–2019 (Eriksson et al. unpublished), the isolates included in our study showed low adhesion capacity, and the cells were rather unresponsive to both carriage and IMD isolates in our experimental model, findings supported by our previous study using an in vivo murine model [30]. The only positive correlation between invasive isolates and increased adhesion observed here was in the B isolates, where IMD isolates also induced increased cell death and proinflammatory activation in the cells. However, an overall analysis of the 22 isolates used in the current study found that adhesion to cells does not strongly correlate with any of the inflammatory markers or cell death. In the outbreak isolates, adhesion was more pronounced in the carriage isolates than in the IMD isolates. This is line with the results from a previous study also using the isolates from outbreak 2 [29], which showed a higher adherence to a different cell line of human pharyngeal cells (Detroit 562 cells) in the carriage isolate. In the previous study it was shown that the invasive isolate produced more SiaA and subsequently more capsule compared to the linked carriage isolate, but also displayed higher expression of PilE and Opa proteins compared to the linked carriage isolate. Two genes (NEIS033 and NEIS2099) possibly involved in adhesion were lacking in the carriage isolate in the genomic comparison made in the present study of the isolates in outbreak 2, which could also partially explain the differences seen in adhesion.

Two recent studies report the highest prevalence of carriage isolates belonging to serogroups Y and B [6, 48]. Interestingly, these groups represent the two with the most opposite cellular responses, with the strongest and weakest induction of adhesion, cell death and proinflammatory activation in our study. In addition, another study comparing invasive and carriage isolates of the ST-4821 complex showed differences in the cellular inflammatory response between carriage and invasive isolates, similar to our findings with serogroup B isolates [49]. Although much available data regarding serogroup B isolates point to a stronger cellular activation upon exposure to invasive isolates than carriage isolates, the other groups are not as clear cut. In our study, isolates are grouped according to serogroups but other approaches such as ST-grouping may be considered. Deghmane et al. used a subset of ST11 isolates describing that the 10 invasive isolates induced apoptosis to a larger extent than the 8 carriage isolates [43].

### Limitations of the study

Although being a relevant and commonly used epithelial cell model to study host–pathogen interactions between meningococci and the host, the experimental design can only address how cells respond to the differences in the bacterial isolates alone. No conclusions on inter-individual variations and factors that may make certain individuals more prone to suffer invasive meningococcal disease upon colonization can be discussed.

In the current study the included isolates are derived from healthy carriage or invasive isolates derived from the reference laboratory repository. Thus, the isolates are, however diverse, potentially virulent and highly clinically relevant. A larger cohort would be required to address generalizable conclusions regarding isolate groups. Thus, in our limited selection we cannot make generalized conclusions based on our data containing only 22 isolates. However, the data indicates that there is no clear correlation between the cellular responses and isolate origin (carriage or IMD isolate) indicating that despite eventual correlations that could be found in big cohorts, these would not be universally applicable to the question regarding host response differences towards carriage vs IMD isolates in the current experimental design. Further detailed studies to decipher the key virulence mechanisms and whether it is the host or the bacteria being the main mediator to discern healthy carriage from invasive disease upon initial host–pathogen interaction are required.

## Conclusions

In conclusion, we aimed to describe the cell-mediated mechanisms potentially involved in the intricate interplay between pathogens and hosts that mediates the colonization and healthy carriage of meningococci. The stronger proinflammatory response found in cells exposed to serogroup B compared to Y. Interestingly, the cellular response to the genetically similar outbreak serogroup C and W isolates showed that the strongest proinflammatory, and thus potentially immunomodulating response, was mounted upon recognition of carriage isolates and not the IMD isolates. Our data indicate that the IMD-associated isolates are not the most potent in inducing proinflammatory activation. Further investigations are required to detail the mechanisms involved, but it is clear that isolate selection is crucial for sustainable conclusions to be drawn in studies of host‒pathogen interactions, especially in the quest to discern the determining factors involved in the development of IMD or the carriage of N*. meningitidis.*

## Methods

### Bacterial isolates and culture

Twenty-two meningococci isolates belonging to serogroup groups B (*n* = 3), C (*n* = 5), W (*n* = 10), and Y (*n* = 4) previously isolated from patients or healthy carriers kept at the National Reference Lab for *Neisseria meningitidis* at Örebro University Hospital, Örebro, Sweden, were included in the study. Serogroup was determined upon initial collection of the isolate using Difco Neisseria Meningitidis Antisera (BD Diagnostics, Sparks, MD) according to the manufacturer instructions before inclusion. Prior to experiments, isolates were grown on GC agar (3.6% Difco GC Medium Base agar (BD Diagnostics, Sparks, MD) supplemented with 1% hemoglobin, 10% horse serum and 1% IsoVitalex (BD Diagnostics)) overnight at 37 °C in a 5% CO_2_ atmosphere prior to each experiment. Colonies were harvested and suspended in PBS, and the bacterial density was set to 2.5 × 10^8^/mL, as determined by OD_600_ using a BioPhotometer (Eppendorf, Hamburg, Germany). Bacterial isolates included in the study were selected to represent capsular groups from asymptomatic carriers and invasive cases as epidemiologically linked groups of cases and carriers (Table 1). Some isolates were from outbreaks where IMD isolates and carriage isolates were linked as described in Table 1. Outbreak 1 was derived from teenagers associated with an outbreak at a World Scout Jamboree whereof one is IMD and three are carriage isolates [32], outbreak 2 was derived from an outbreak of invasive meningococcal disease in the French Alps whereof one is IMD and one is a carriage isolate [29] and outbreak 3 was derived from a local outbreak whereof all three are IMD isolates [31]. IMD isolates were collected from blood and the linked carriage isolates from nasopharyngeal swabs. Phylogenetic trees using the genome comparator tool in PubMLST [50] of all isolates (Supplementary Figure 1a) and outbreak isolates exclusively (Supplementary Figure 1b) shows the genetic relatedness of previously whole-genome sequenced isolates as per reference in Table 1.

### Cell culture and infection experimental setup

Pharyngeal epithelial cells (FaDu: HTB-43™) were maintained in EMEM (ATCC, Manassas, VI) with 10% FBS (Gibco, Thermo Fisher Scientific, Waltham, MA) and 100 U/mL penicillin‒streptomycin (Gibco) in tissue culture flasks (Sarstedt, Nürnbrecht, Germany), and upon confluence, the cells were detached by trypsin (Gibco) and reseeded. Twenty-four hours before the experiments, the cells were trypsinized and suspended in EMEM with 2% FBS and seeded in a 24-well plate at a density of 3 × 10^5^ cells per well. One hour prior to the experiment, the medium was changed to fresh EMEM with 2% FBS. Bacteria suspended in EMEM with 2% FBS were added to the cells at a multiplicity of infection (MOI) of 10, and the plate was placed on a plate shaker at 200 rpm for 1 min to evenly distribute the bacteria. Following 6 h of incubation at 37 °C in a 5% CO_2_ atmosphere, supernatants were collected, and cells were washed twice in PBS and once in EMEM with 2% FBS and gentamycin, with each wash including shaking the plate at 200 rpm for 1 min. Following washes, the cells were further incubated in EMEM with 2% FBS and gentamycin at 37 °C in a 5% CO_2_ atmosphere. Supernatants were collected following an additional 18 h incubation. Collected supernatants were centrifuged at 5000 × g for 5 min, aliquoted and stored at -80 °C until analysis. Six biological replicates (separate cell and bacteria preparations on separate days) with three technical repeats (identical experimental conditions in wells on the same plate) were performed for each isolate.

### Adhesion of meningococci to epithelial cells

To understand how the level of host-microbe interaction affects the outcome, bacterial adhesion to the epithelial cells was quantified using a modified CFU protocol [51]. For determination of cell-associated bacteria following the initial 6 h incubation, culture media was removed, and cells were washed three times in warm PBS using a plate shaker at 200 rpm for 1 min followed by cell lysis with 1% saponin in PBS for 15 min. Lysates were collected, rigorously pipetted to disrupt any remaining cells, serially diluted in PBS and plated on GC agar. Plates were incubated overnight at 37 °C in a 5% CO_2_ atmosphere and photographed, and CFUs were counted manually using ImageJ software. Five biological replicates (separate cell and bacteria preparations on separate days) containing three technical repeats (three experimental wells per plate), plated in triplicate, were performed for each isolate (i.e., each isolate resulted in 45 CFU measurements).

### Cytokine quantification and cell viability assay

Cytokines were quantified using the LEGENDplex Human Inflammation Panel analyzing CCL2, CXCL8, IFN-α, IFN-γ, IL-1β, IL-6, IL-10, IL-12p70, IL-17A, IL-18, IL-23, IL-33 and TNF (BioLegend, San Diego, CA) on an Accuri C6 (Becton Dickinson, San Jose, CA) according to the manufacturer’s instructions. Cell viability was assessed by adding CellTiter-Blue® (Promega, Madison, WI) to the cells following the final 18 h collection of supernatants, and fluorescence was read on a BioTek® Synergy H1 (Agilent, Santa Clara, CA) according to the manufacturer’s instructions.

### Statistics

Differences between groups of isolates were calculated on the means (of all technical replicates) from each biological replicate with one-way ANOVA using Kruskal‒Wallis testing and Dunn’s multiple comparison corrections or Mann‒Whitney test between carriage and IMD isolates within serogroups. The correlation between CFU/cell and cellular response parameters was calculated using Spearman’s rank correlation coefficient.

### Supplementary Information

**Supplementary Material 1. **

**Supplementary Material 2. **

## Data Availability

The datasets used and/or analysed during the current study are available from the corresponding author on reasonable request.
