# Supplementary material for: Diverse proinflammatory response in pharyngeal epithelial cells upon interaction with Neisseria meningitidis carriage and invasive isolates
Source: BMC Infect Dis. 2024 Mar 6;24:286. doi: 10.1186/s12879-024-09186-3 (PMC10916014; doi:10.1186/s12879-024-09186-3)
Supplement: Supplementary file 1 — Supplementary Material 1. [file 12879_2024_9186_MOESM1_ESM.pdf]

a)

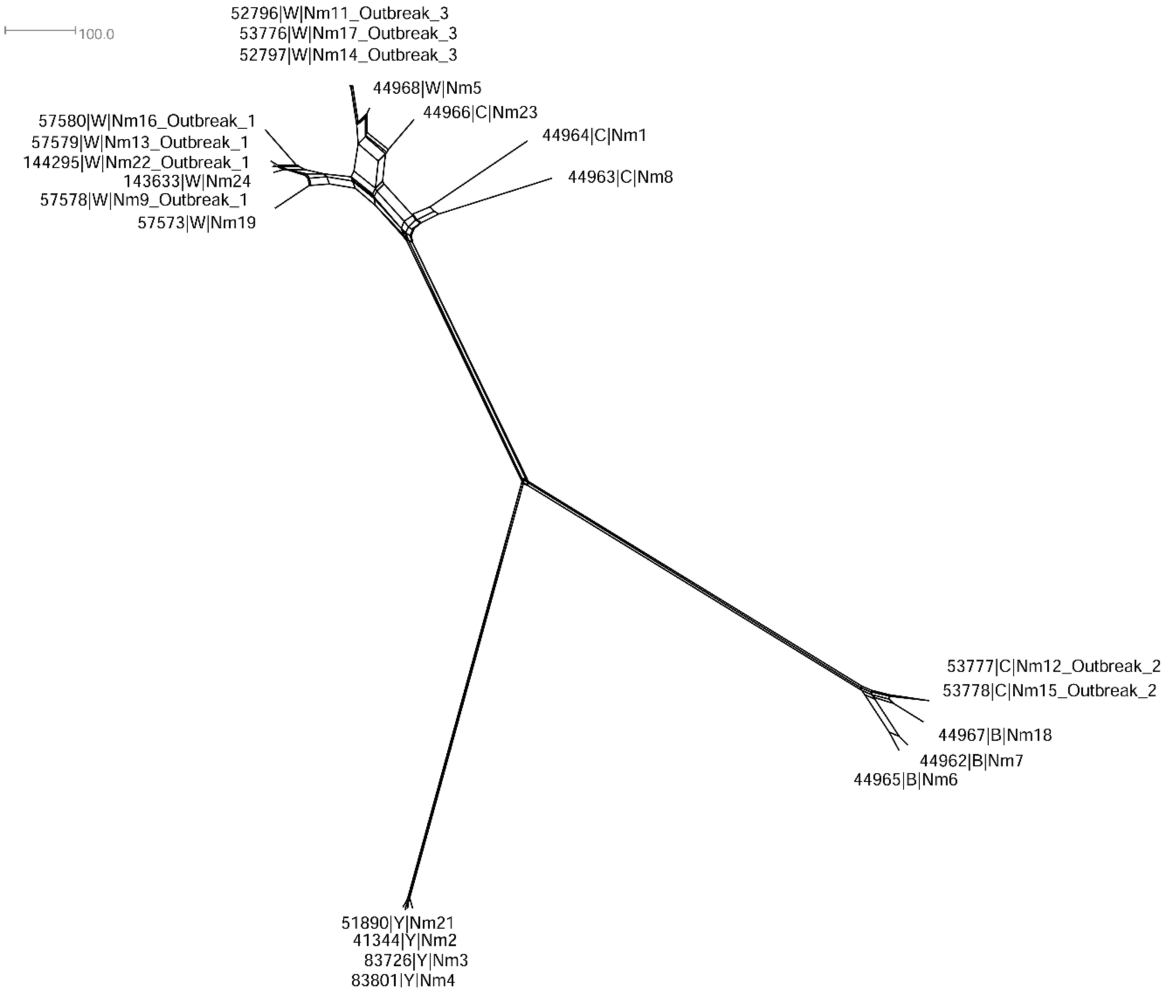
100.0
52796|W|Nm11_Outbreak_3
53776|W|Nm17_Outbreak_3
52797|W|Nm14_Outbreak_3
44968|W|Nm5
44966|C|Nm23
57580|W|Nm16_Outbreak_1
57579|W|Nm13_Outbreak_1
144295|W|Nm22_Outbreak_1
143633|W|Nm24
57578|W|Nm9_Outbreak_1
57573|W|Nm19
44964|C|Nm1
44963|C|Nm8
53777|C|Nm12_Outbreak_2
53778|C|Nm15_Outbreak_2
44967|B|Nm18
44962|B|Nm7
44965|B|Nm6
51890|Y|Nm21
41344|Y|Nm2
83726|Y|Nm3
83801|Y|Nm4

b)

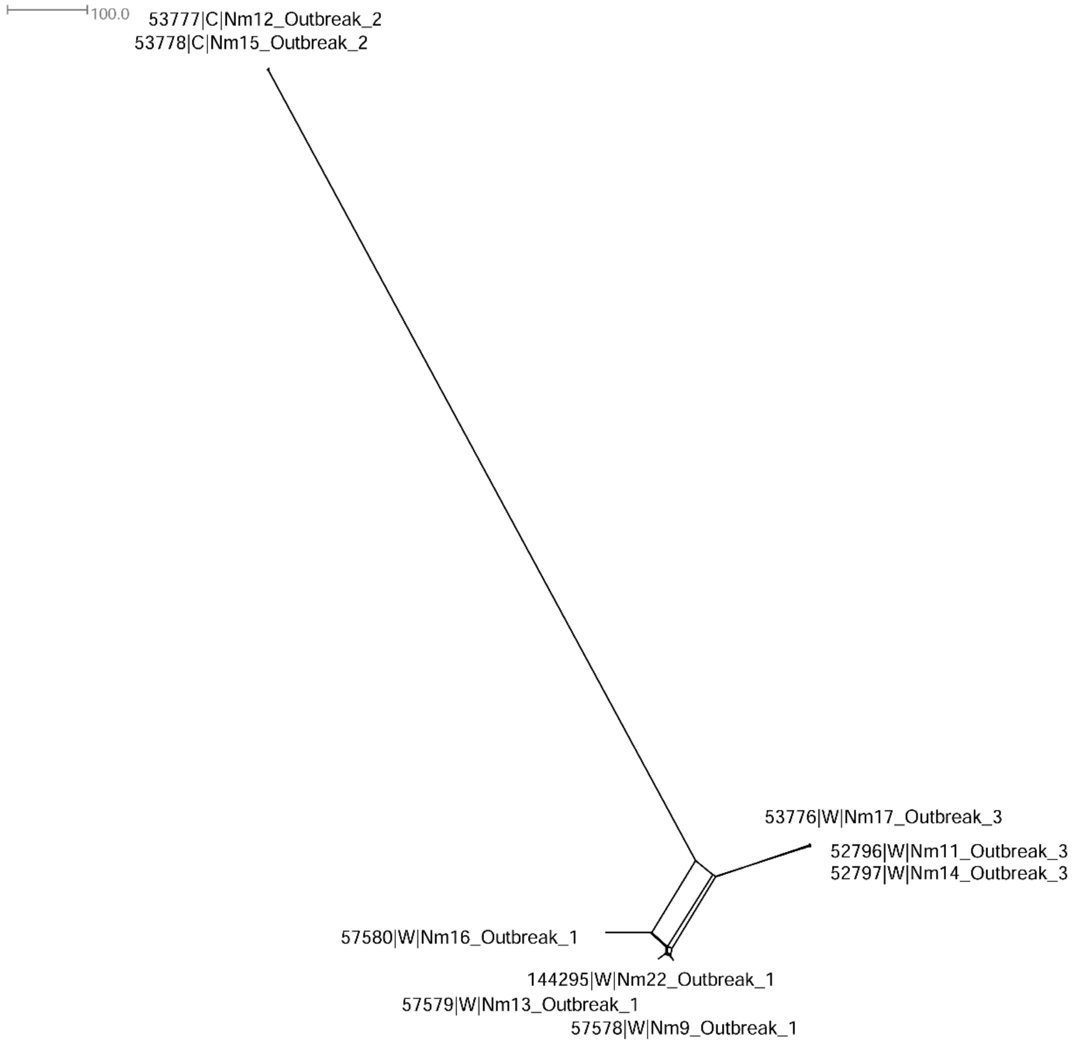

Supplementary Figure 1. *Neisseria meningitidis* genome comparison. Neighbornet network showing the genomic relatedness of a) all isolates and b) outbreak isolates in 1,422 core loci of *Neisseria meningitidis* v2 (Jolley KA, Bray JE, Maiden MCJ. Open-access bacterial population genomics: BIGSdb software, the PubMLST.org website and their applications. Wellcome Open Res. 2018;3:124). PubMLST ID, capsule group, alias and outbreak designation (if any) is shown for each node.
