# Supplementary material for: Diverse proinflammatory response in pharyngeal epithelial cells upon interaction with Neisseria meningitidis carriage and invasive isolates
Source: BMC Infect Dis. 2024 Mar 6;24:286. doi: 10.1186/s12879-024-09186-3 (PMC10916014; doi:10.1186/s12879-024-09186-3)
Supplement: Supplementary file 2 — Supplementary Material 2. [file 12879_2024_9186_MOESM2_ESM.pdf]

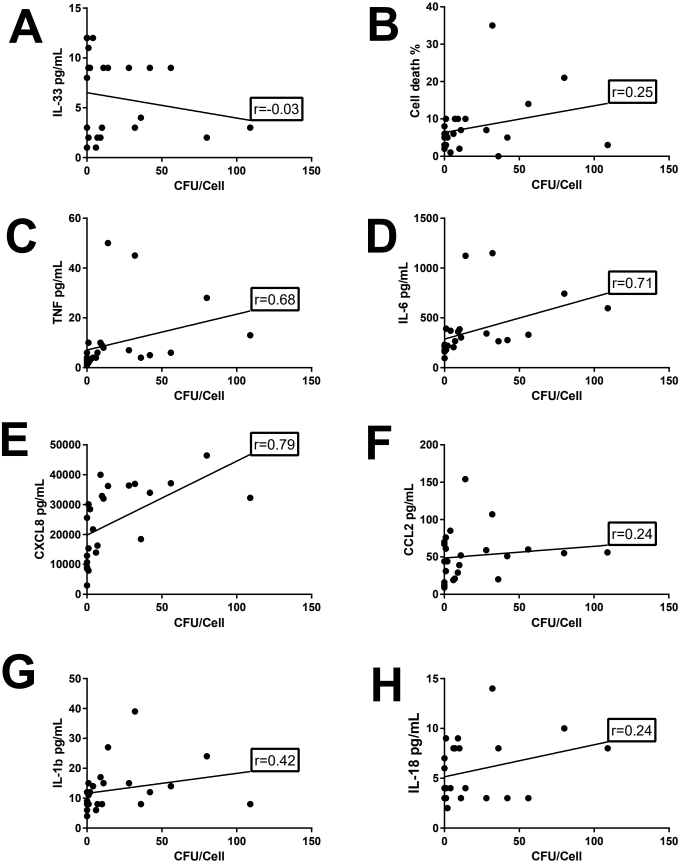

Supplementary figure 2. Correlation graphs for adhesion vs A: IL-33, B: cell death, C: TNF, D: IL-6, E: CXCL8, F: CCL2, G: IL-1b and H: IL-18 for all isolates included in the study.
